# Regression of hepatic fibrosis in gamma-irradiated rats by mycophenolate mofetil: potential role of Egr-1

**DOI:** 10.1007/s00210-025-04287-5

**Published:** 2025-06-17

**Authors:** Shereen Mohamed Galal, Dalia Mohamed Mostafa, Shereen Mohamed El kiki

**Affiliations:** 1 Health Radiation Research Department, National Center for Radiation Research and Technology, Egyptian Atomic Energy Authority (EAEA), Cairo, Egypt; 2 Radiation Biology Department, National Center for Radiation Research and Technology, (NCRRT), Egyptian Atomic Energy Authority (EAEA), Cairo, Egypt

**Keywords:** Mycophenolate mofetil, Gamma irradiation, Early growth response 1, Liver fibrosis

## Abstract

One of the prominent adverse consequences of radiation treatment to the upper right quadrant is hepatic fibrosis. There is an argument that early growth response 1 (Egr-1) manages the hepatic stellate cell activation. Mycophenolate mofetil (MMF), a potent immunosuppressive medication that exhibits potent anti-inflammatory, anti-apoptotic, and antioxidant effects, is frequently prescribed to prevent organ transplant rejection. The purpose of this investigation is to explore the molecular pathways that underpin radiation-induced hepatic fibrosis and to assess the potential influence of MMF on Egr-1 over radiation (IRR)-induced fibrogenesis in rats. MMF (100 mg/kg b.wt. oral) was given to rats exposed to IRR for 4 weeks in a row at a total dose of 12 Gy divided into four doses of 3 Gy each. Assessment of the data demonstrated that MMF administration repressed Egr-1 signaling induced by IRR, diminished oxidative stress, strengthened antioxidant defenses, lessened hepatic inflammatory responses, and suppressed the production of pro-fibrogenic cytokines. Additionally, MMF therapy repaired the histopathological changes brought on by IRR as well as allowed the liver’s normal architecture to be restored. In conclusion, the evidence collected revealed that MMF effectively enhanced the architecture of the fibrotic liver and ameliorated the biochemical indicators of liver fibrosis by inhibiting Egr-1.

**Graphical Abstract:**

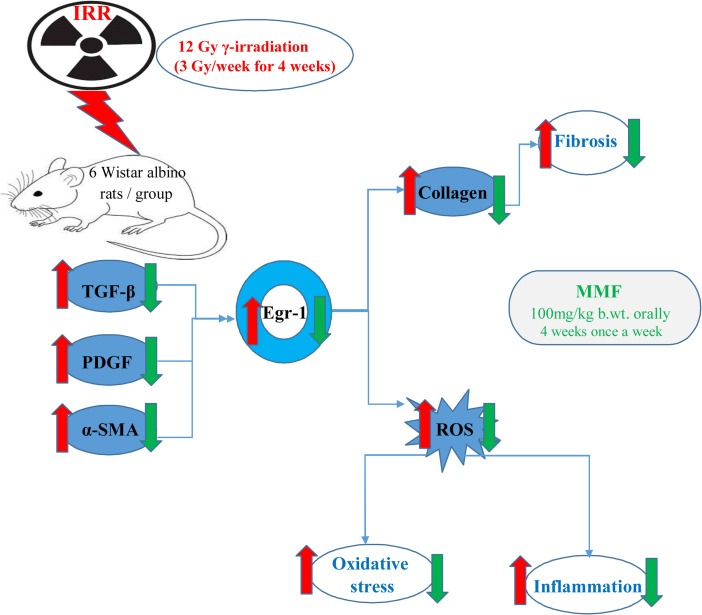

## Introduction

The overabundance of extracellular matrix (ECM) proteins resulting from persistent inflammation and hepatic cell death in most chronic liver disorders is known as hepatic fibrosis (Bataller and Brenner 2005; Jiao et al. 2009). Liver fibrosis can result from numerous reasons, including the hepatitis viruses, alcohol, medications, and chemical reagents. When liver fibrosis persists, the accumulated ECM damages the normal architecture of the liver by creating a fibrous scar. This leads to the development of cirrhosis, a disorder characterized by severe liver scarring that impairs liver function and restricts hepatic blood flow, necessitating liver transplantation in many cases (Lee and Friedman 2011).

While early growth response 1 (Egr-1) has long been considered to be an important mediator of cell growth, differentiation, and apoptosis, recent studies expand on the spectrum of the recognized biological activities associated with this transcription factor by highlighting its potential role in physiological tissue repair and pathological fibrosis (Woodson and Kehn-Hall 2022). Egr-1 expression is induced in a variety of cell types by transforming growth factor-β (TGF-β) and other fibrogenic stimuli and, in turn, Egr-1 directly stimulates the production of collagen, matrix accumulation, reactive oxygen species (ROS) generation, and myofibroblast differentiation, as well as the secretion of TGF-β, platelet-derived growth factor (PDGF), cytokines, and other fibrogenic growth factors in response to ionizing radiation (Li et al. 2003; Bhattacharyya et al. 2013). Each of these factors contributes to the development and persistence of fibrosis. Fibrotic tissues in a variety of human diseases and animal models show increased Egr-1 accumulation in affected organs, and evidence of active Egr-1-dependent signalling. Indeed, the presence of the “Egr-1 signature” potentially predicts more rapid fibrosis progression (Bhattacharyya et al. 2013) which is significantly increased by radiation *via* activation of hepatic stellate cells (HSCs).

Radiation’s direct and indirect impacts initiate a series of molecular and biochemical signalling processes that have the potential to either reverse the damage or cause irreversible physiological alterations or death of cells (Spitz et al. 2004; Azzam et al. 2012). The majority of liver pathogenic disturbances may result in oxidative stress, which can cause DNA damage, lipid peroxidation, and protein oxidation. This can impair hepatocyte mitochondria, exacerbate inflammation, and trigger the onset of fibrosis (Sánchez-Valle et al. 2012).

Oxidative damage is indicated by the formation of lipid peroxidation byproducts that rise with increasing radiation doses in the liver of rats (Mansour et al. 2008). Dawson et al. (2002) reported that radiation-induced liver damage, with restricted treatment choices, can lead to liver failure and death in extreme cases.

MMF is transformed into its active metabolite, mycophenolic acid. This reversible, non-competitive inhibitor of inosine monophosphate dehydrogenase is commonly used to avoid organ transplant rejection (Staatz and Tett 2014). MMF reduces the amounts of guanosine triphosphate in monocytes and lymphocytes and inhibits the synthesis of new purines, hence inhibiting their proliferation. MMF also has anti-inflammatory properties under inflammatory stress (Ysebaert et al. 2003; Huang et al. 2018). MMF’s biological effects have been demonstrated in recent years to be more than just immunosuppressive and exhibit antitumoral characteristics, especially in cancer cells of the colon and prostate, through protein adhesion and this is crucial for the spread and recurrence of tumors (Engl et al. 2005). Other researchers found that MMF treatment inhibits ROS and/or NRS over generation in intestinal cancer cells and endothelial function in rats, which has an antioxidant impact both in vitro and in vivo (Fréguin-Bouilland et al. 2011; Fernandes et al. 2014; Ferjani et al. 2015).

Bhattacharyya et al. (2013) reported that identifying molecules and pathways that are abnormally expressed or activated during fibrogenesis opens the way for creating targeted techniques to reduce fibrosis. Based on this point of view, the current study proposed that MMF showed potent inhibitory effects on Egr-1 induction or activity. It could be a novel and promising approach for radiation-induced liver fibrosis prevention.

## Materials and methods

### Materials

MMF was obtained from Roche Co. dissolved in 0.9% saline (Ferjani et al. 2016). All of the other chemicals utilized in this investigation were of analytical quality.

### Experimental animals

The National Center for Radiation Research and Technology provided male Wistar albino rats of 6 weeks weighing (130–150 g). Animals were kept in normal temperature and humidity conditions for the experiment settings (22–24 °C), 55 ± 10% humidity, and a 12/12-h light/dark cycle. The recipient received regular concentrated food pellets that contained all the necessary nutrients (23% protein, 4.68% lipids, and 2.6% fiber) with unrestricted access to water. The Animals (Scientific Procedures) Act, 1986 in the UK, as well as the EU Directive 2010/63/EU for animal research, were followed in the conduct of this investigation and the regulations of the animal care ethical committee at the National Center for Radiation Research and Technology (NCRRT), Cairo, Egypt. There was a concerted effort to lessen animal suffering.

### Radiation facility

Rats were exposed to whole-body radiation at the NCRRT in Cairo, Egypt, using a Gamma cell-40 equipped with a Caesium-137 irradiation source. Rats were exposed to four doses of γ-irradiation (3 Gray(Gy)/week for 4 weeks) (Hasan et al. 2017) administered at a rate of 0.012 Gy/s.

### Experimental setup

Four groups of rats were divided into the following: (i) control group (C)—rats received 0.5 ml saline orally once/week for 4 weeks; (ii) MMF—rats treated with 0.5 ml of MMF (100 mg/kg b.wt. orally) for 4 consecutive weeks once a week (Beduschi et al. 2013); (iii) irradiated group (IRR)—rats exposed to whole-body radiation using four fractions of γ-irradiation (3 Gy for each fraction, up to a total dose of 12 Gy within 4 weeks); (iv) irradiation + mycophenolate mofetil group (IRR+MMF)—rats were exposed to γ-irradiation (four fractions; 3 Gy for each fraction), up to a total dose of 12 Gy within 4 weeks concurrently with MMF at (100 mg/kg b.wt.) for 4 consecutive weeks. A day following the last dosage of MMF, animals (each group contains six rats) were sacrificed in a state of mild anesthesia using urethane (1.2 g/kg) (Field et al. 1993); blood samples were obtained by heart puncture then serum was separated by centrifugation and liver samples were immediately removed, rinsed with saline to eliminate blood contamination, dried by blotting with filter paper, and then divided into two portions for various biochemical and histopathological examinations.

### Body mass and liver mass index

Rats’ body mass was measured in the morning using a digital balance at the start of the experiment and after the course of the experiment (4 weeks later). Liver mass ratios were assessed by thoroughly excising the livers and weighing them following animal sacrifice. The ratio was calculated using the following equation (Al-Attar and Zari 2010),

$$\text{Liver mass index }({\%}) =\text{ Liver mass }(\text{g})/\text{Body mass }(\text{g}) \times 100$$

### Western blot

Proteins were extracted from liver tissue homogenates using ice-cold radioimmunoprecipitation assay (RIPA) buffer supplemented with phosphatase and protease inhibitors (50 mmol/L sodium vanadate, 0.5 mM phenylmethylsulphonyl fluoride, 2 mg/mL aprotinin, and 0.5 mg/mL leupeptin), then centrifuged at 15,000 *g* for 20 min. The protein concentration for each sample was determined using Bradford assay (He 2011). Equal amounts of protein (20–30 µg of total protein) were separated by SDS/polyacrylamide gel electrophoresis (10% acrylamide gel Cat # 161-0181) using a Bio-Rad Mini-Protein II system. The protein was transferred to polyvinylidene difluoride membranes (Pierce, Rockford, IL, USA) with a Bio-Rad Trans-Blot system. Transfer of protein from gel to membrane was confirmed by using Ponceau red stain; the membranes were washed with PBS and were blocked for 1 h at room temperature with 5% (w/v) skimmed milk powder in PBS. The manufacturer’s instructions were followed for the primary antibody reactions. Following blocking, the blots were developed using antibodies for early growth response 1 (Egr-1) (1:1000; Cell Signaling technology; USA, Cat. # 4153), alpha smooth muscle actin** (**α-SMA) (1:1000; Cell Signaling technology; USA, Cat. # 14968), TGF-β (1:1000; Santa Cruz Biotechnology; USA, Cat. # sc-130348), PDGF (1:1000; Invitrogen Thermo Fisher Scientific; USA, Cat. # PA5-17753), and β-actin (1:1000; Sigma; Cat. # A1978). The membrane was incubated overnight at pH 7.6 at 4 °C with gentle shaking. After washing, peroxidase-labeled secondary antibodies were added, and the membranes were incubated at 37 °C for 1 h. Band intensity was analyzed by a ChemiDocTM imaging system with Image LabTM software version 5.1 (Bio-Rad Laboratories Inc., Hercules, CA, USA). The results were expressed as arbitrary units after normalization for β-actin protein expression.

### Biochemical assay

#### Hepatotoxicity biomarkers

All photometric determinations were done using (Thermo Electron UV-Visible spectrophotometers U.S.A.). In serum, the activity of aspartate amino-transferase (AST), alanine amino-transferase (ALT), and gamma glutamyl transferase (GGT) was determined according to Lala et al. (2023). Total protein (TP) was determined according to the procedures described by Simonian and Smith (2006), using BioSystems kit (Spain).

#### Oxidative stress indices

In liver tissue*,* NADPH oxidase-4 (NOX-4, LifeSpan biosciences, Cat. No: LS-F17464-1), nuclear factor erythroid–related factor-2 (Nrf2, Northwest, Cat. No: NFE2L2), and hemoxygenase-1 (HO-1, BioVision, Cat. No: E4525-100) protein were detected by ELISA Kits, using Elisa microplate reader (DV 990 BV 416; Gio.DE VITA and CO., Rome, Italy), respectively, according to the manufacturer’s procedure. The glutathione peroxidase (GPx) activity was determined photometrically, as described by Chafik et al. (2018). Furthermore, hepatic nitric oxide (NO) level was indirectly calculated using the nitrite/nitrate concentration approach (Miranda et al. 2001).

#### Inflammatory mediators

ELISA kits (MyBioSource.com, Inc., San Diego, USA) were used to estimate the contents of hepatic tumor necrosis factor-α (TNF-α, Cat. No: MBS175904), nuclear factor kappa-B (NF-κB, Cat. No: MBS3809785), and interleukin-6 (IL-6, Cat. No: MBS2021530) following the manufacturer’s procedure.

### Histopathological examination

All animal groups’ liver tissue samples were gathered and preserved in 10% neutral buffered formalin. The fixed specimens were then trimmed, washed, and dehydrated in increasing grades of alcohol, cleaned in xylene, embedded in paraffin, sectioned at 4–6-μm thickness, and stained with hematoxylin and eosin, as described by Bancroft and Gamble (2008). Sections of prepared slides were inspected using a light microscope (Olympus xc30, Tokyo, Japan).

Semi-quantitative analysis was used to determine the incidence and severity of liver lesions, as described by Hayes and Kobets (2023) using a scale where, grade 0: no apparent injury; grade I: swelling of hepatocytes; grade II: ballooning of hepatocytes; grade III: lipid droplets in hepatocytes; and grade IV: necrosis of hepatocytes.

Masson’s trichrome staining was used to assess the collagen levels in the liver tissue.

The following criteria were used to assign a numerical grade to the liver sections’ degree of fibrosis: 0, no fibrosis; I, a slight amount of fibrosis, fibrosis located in the central hepatic lobule; II, mild fibrosis, widened central fibrosis; III, extreme fibrosis (Park et al. 2016).

### Analysis of data

The statistical program for social science (SPSS) version 21.0 for Windows was used to conduct the analysis, which included one-way analysis of variance (ANOVA) and Duncan’s multiple range test. The level of significance was determined using *P* values of 0.05. The standard error of the mean (SEM) is used to represent values.

## Results

### Impact of MMF administration on fibrotic rats’ body mass gain and liver mass index

**Fig. 1:**
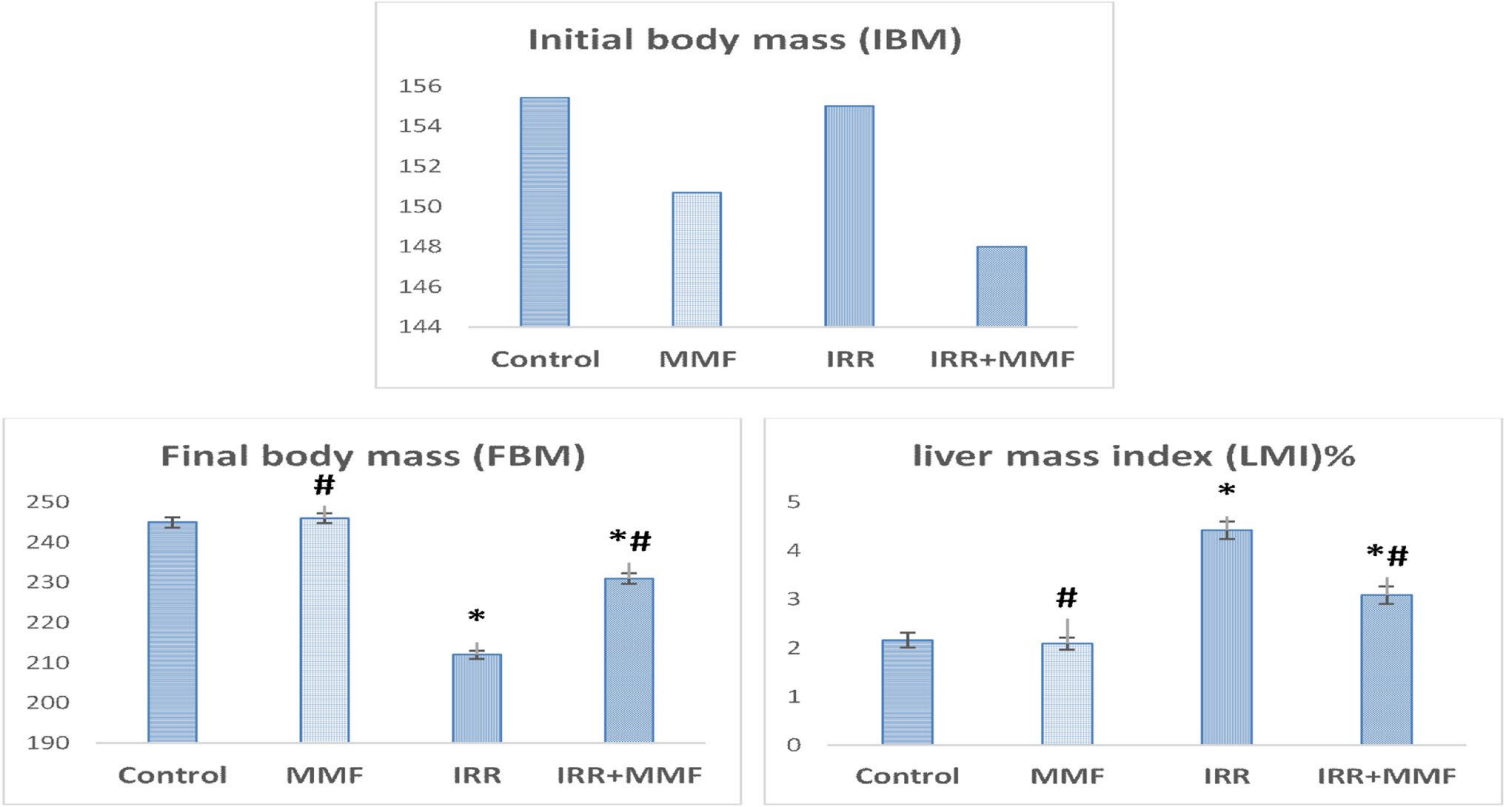
Effect of MMF treatment on body mass gain and liver mass index of fibrotic rats. Data are presented as mean ± SEM, *n* = 6. * and # indicate significant changes from control and IRR respectively at *P* < 0.05 using ANOVA followed by Duncan as a post ANOVA test

Figure 1 illustrates that the IRR group exhibited a substantial rise (*P* < 0.05) in the liver mass index (%) plus a significant reduction (*P* < 0.05) in body mass gain in comparison with the control group. Comparing the MMF group to the control group, there was no discernible difference in body mass gain or liver mass index. Furthermore, in contrast to the IRR group, the combination of MMF and IRR led to a rebound of body mass gain and liver mass index (%), indicating the hepato-protection potential provided by MMF treatment as well as a decrease in the hepatotoxicity brought on by radiation therapy.

### MMF administration modulates the expression of Egr-1 and pro-fibrotic biomarkers correlated with activation of hepatic stellate cells in fibrotic rats

**Fig. 2:**
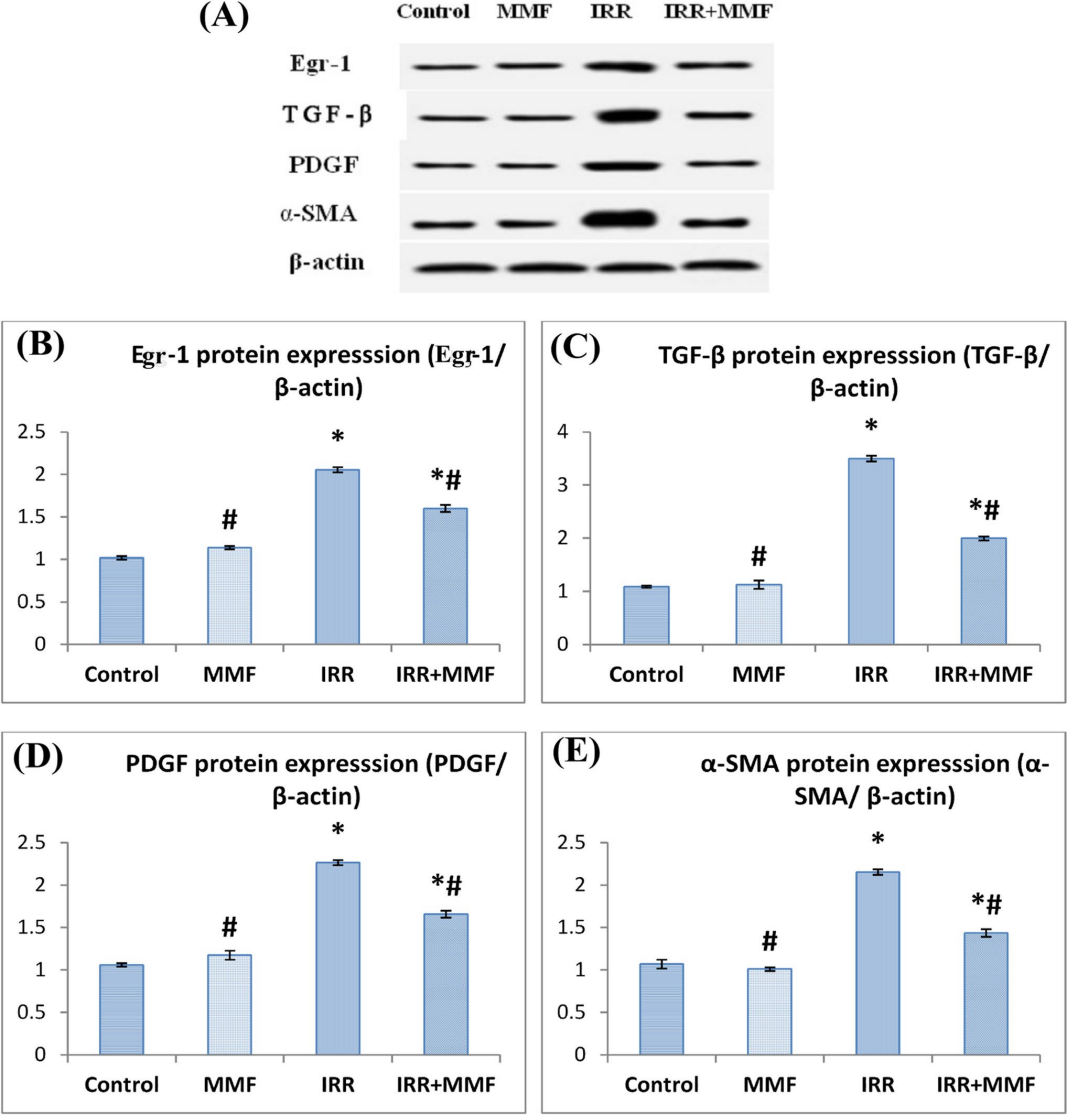
MMF treatment modulates the expression of Egr-1 and pro-fibrotic markers associated with HSC activation in radiation-induced liver fibrosis. Data are presented as mean ± SEM, *n* = 6. * and # indicate significant changes from control and IRR respectively at *P* < 0.05 using ANOVA followed by Duncan as a post ANOVA test. **A** Protein expression by Western blot; **B** Egr-1: early growth response 1; **C** TGF-β: transforming growth factor-β; **D** PDGF: platelet-derived growth factor; **E** α-SMA: alpha smooth muscle actin

Irradiated rats exhibited a noteworthy rise (*P* < 0.0001) in Egr-1 and α-SMA expression of the activated HSC biomarker, additionally, the profibrogenic cytokine TGF-β and PDGF levels in comparison with the control group, while MMF treatment to irradiated rats produced a substantial drop in parameters as mentioned in the above expression in contrast to the irradiated group (Fig. 2).

### Impact of MMF administration on liver function biomarkers in fibrotic rats

**Fig. 3:**
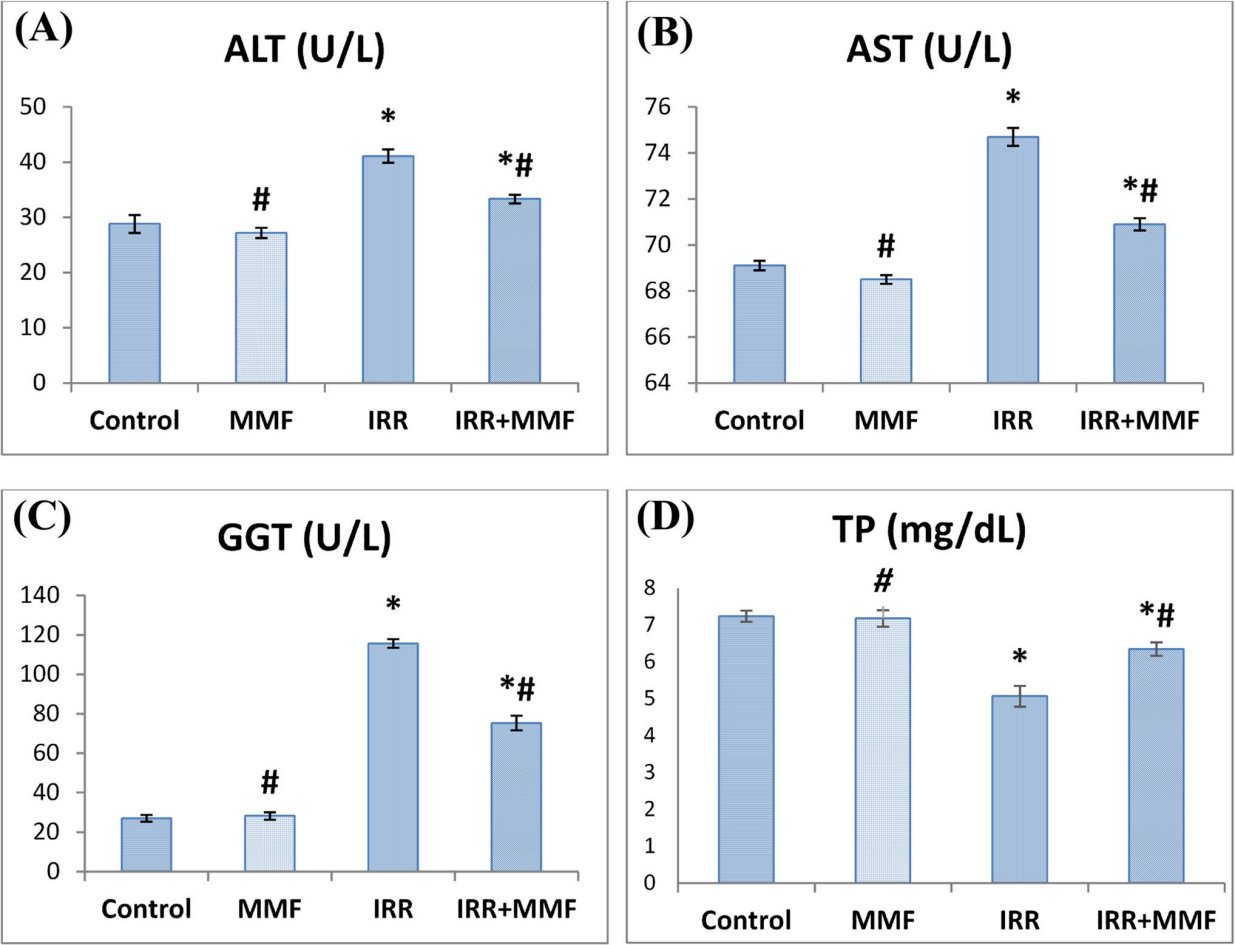
Effect of MMF treatment improves hepatotoxicity biomarkers in fibrotic rats. Data are presented as mean ± SEM,* n* = 6. * and # indicate significant changes from control and IRR respectively at *P* < 0.05 using ANOVA followed by Duncan as a post ANOVA test. **A** ALT: alanine amino-transferase; **B** AST: aspartate amino-transferase; **C** GGT: gamma glutamyl transferase; **D** TP: total protein

Elevated serum activity of ALT, AST, and GGT indicated gamma irradiation, associated with a notable drop (*P* < 0.0001) in the serum level of TP in contrast to the corresponding values observed in the control rats. IRR+MMF-treated group showed significant decrease (*P* < 0.0001) in AST, ALT, and GGT activities. TP serum level was substantially boosted (*P* < 0.0001) after MMF treatment in irradiated rats, indicating that the liver’s synthetic function had been restored (Fig. 3).

### Impact of MMF administration on liver’s antioxidant defenses and oxidative stress

**Fig. 4:**
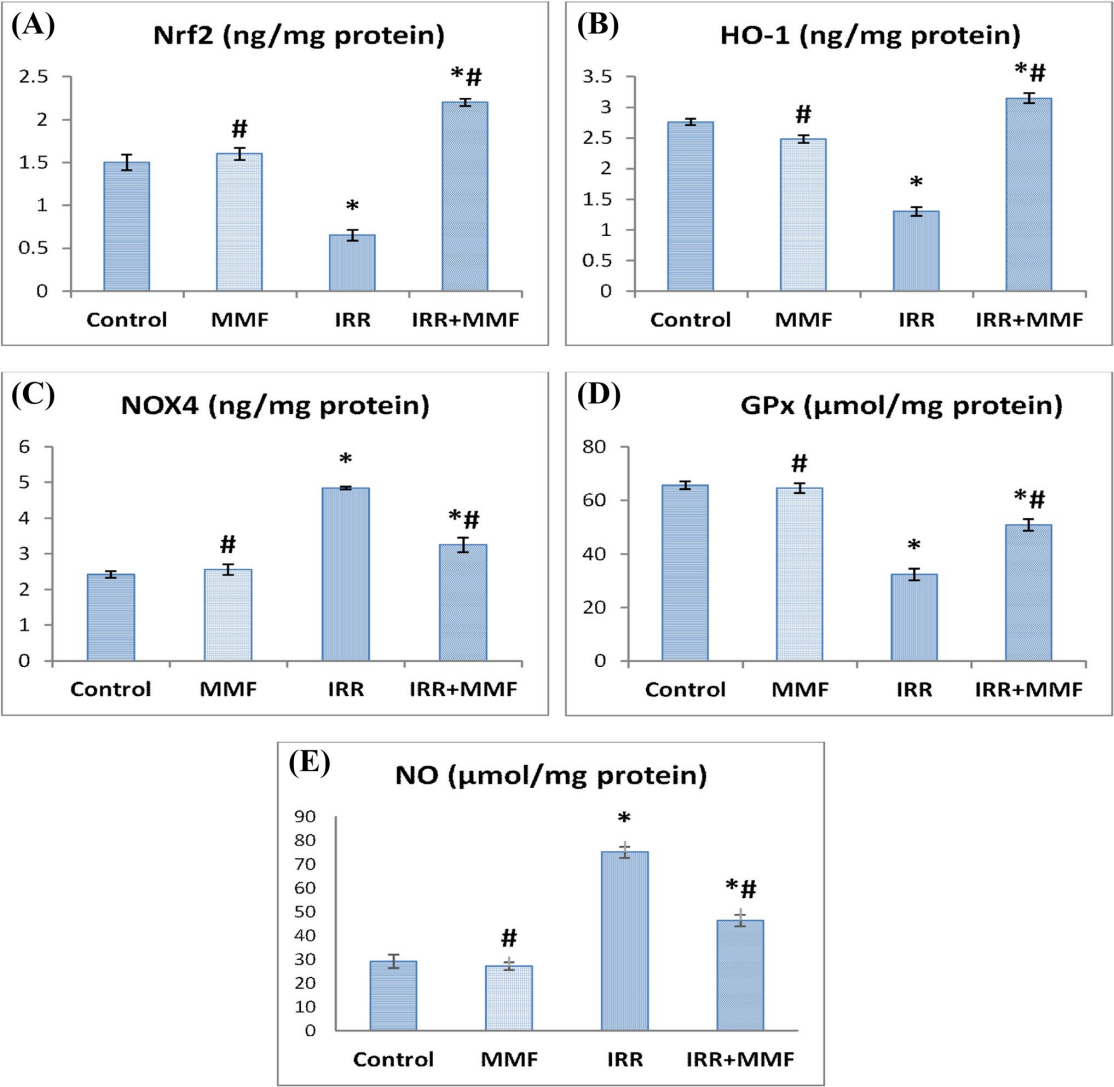
Effect of MMF treatment halts oxidative stress and enhances antioxidant defenses in the liver. Data are presented as mean ± SEM, *n* = 6. * and # indicate significant changes from control and IRR respectively at *P* < 0.05 using ANOVA followed by Duncan as a post ANOVA test. **A** Nrf2: nuclear factor erythroid–related factor-2; **B** HO-1: hemoxygenase-1; **C** NOX4: NADPH oxidase-4; **D** GPx: glutathione peroxidase; **E** NO: nitric oxide

A pronounced spike in NOX4 activity and NO content accompanied with significant depletion (*P*<0.0001) in Nrf2 level, HO-1, and GPx activities in IRR rats compared to the control values and this was modulated by MMF administration leading to a notable elevation in Nrf2 level, HO-1, and GPx activities combined with significant decrease in NOX4 activity and NO content in contrast to irradiated group (Fig. 4).

### Impact of MMF administration on the mediators of inflammation in fibrotic rats

**Fig. 5:**
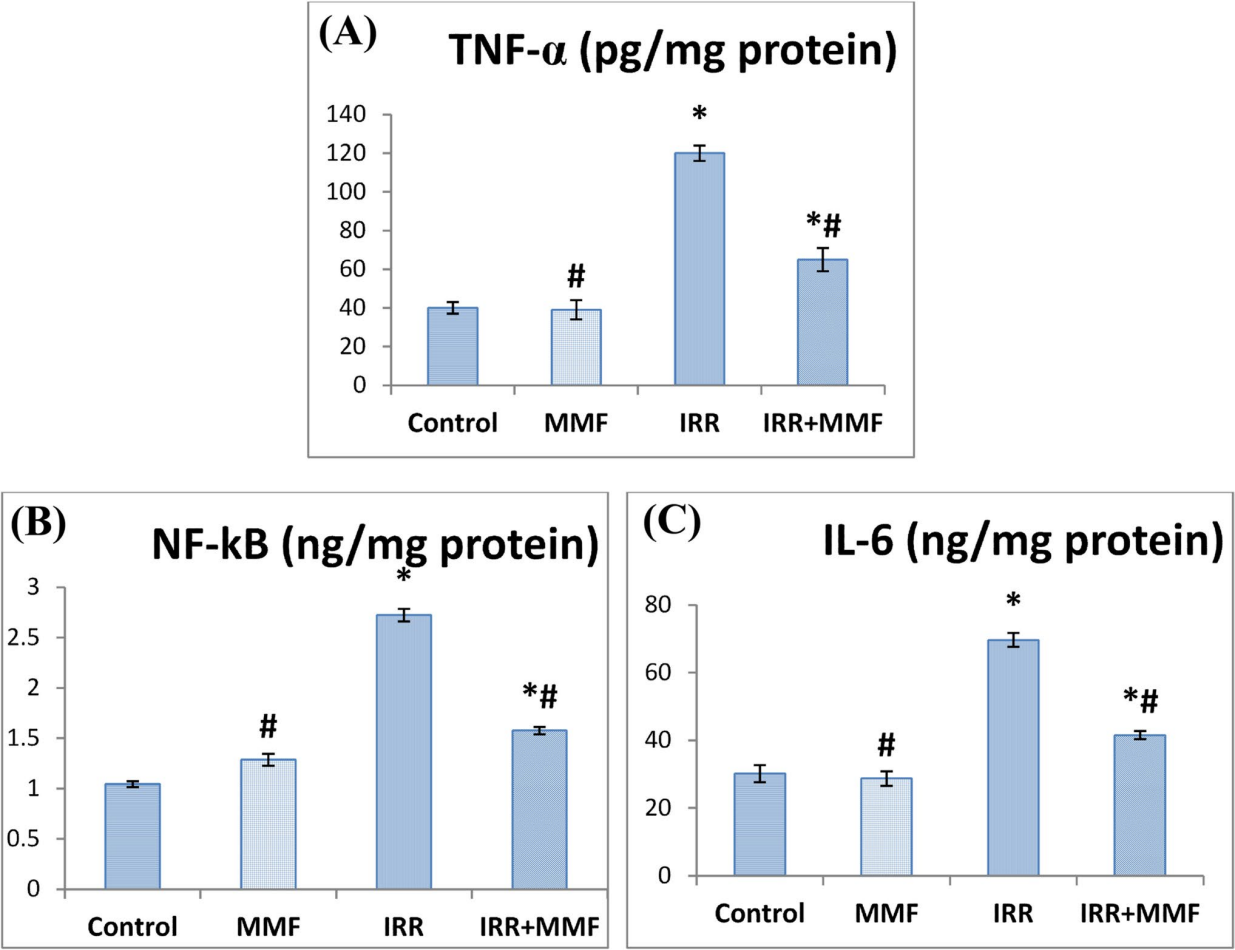
Effect of MMF on inflammatory mediators in radiation-induced liver fibrosis. Data are presented as mean ± SEM, *n* = 6. * and # indicate significant changes from control and IRR respectively at *P* < 0.05 using ANOVA followed by Duncan as a post ANOVA test. **A** TNF-α: tumor necrosis factor-α; **B** NF-κB: nuclear factor kappa-B; **C** IL-6: interleukin-6

Increased levels of pro-inflammatory mediators, including TNF-α, NF-κB, and IL-6, in comparison with the control group, demonstrated the pathophysiology of gamma irradiation. In response to irradiated rats, the IRR+MMF-treated group displayed reduced levels of aforementioned pro-inflammatory mediators (Fig. 5).

### MMF administration and hepatic histology

**Fig. 6:**
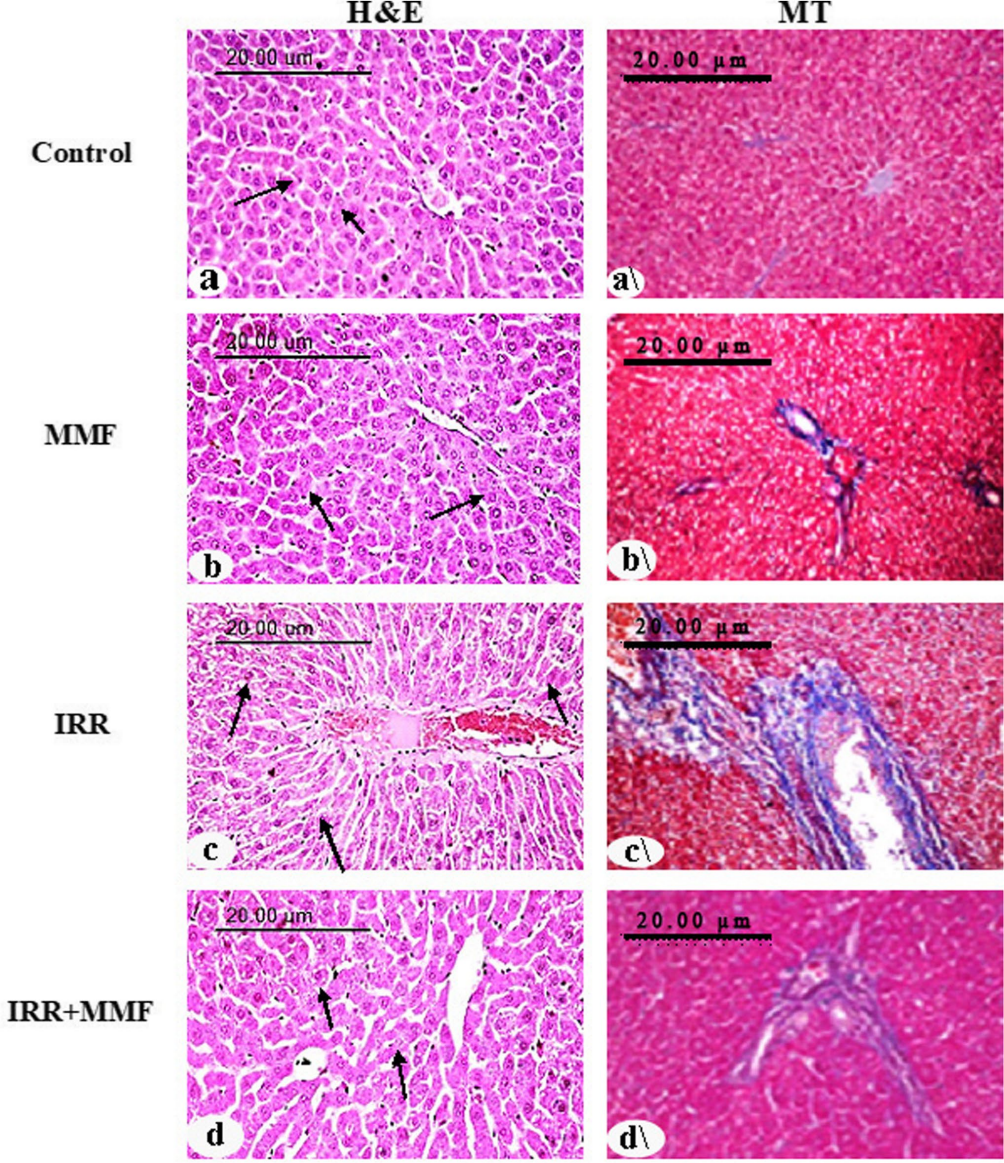
MMF treatment alleviates radiation-provoked histopathological aberrations in liver. (H&E) Left panel: hematoxylin and eosin stain. The histological examination of the liver obtained from control and MMF groups showed normal organization of hepatic cords arrow (**a** and **b**). However, irradiated rats liver revealed nuclear pyknosis and apoptosis of hepatocytes arrow (**c**). The liver of the IRR+MMF group showed mild swelling of hepatocytes arrow (**d**). (H&E × 200). (MT) Right panel: Masson trichrome-stained histopathological tissue section of control and MMF groups showed normal histological structure and delicate fibrous tissues in portal triads (**a**\, **b**\). However, irradiated rats liver showed dilatation of portal vein and fibroplasia (**c**\). The IRR+MMF group showed delicate fibrous tissues in portal triads (**d**\). (MT X200)

The control and MMF groups’ hepatic tissue sections displayed normal hepatic lobule histology and hepatic cord organization, with a large central hepatic vein. Anastomosing plates were used to connect polygonal hepatic cells, with borders facing either the sinusoids or nearby hepatocytes (grade 0). A tissue section stained with Masson’s trichrome showed intact portal triad architecture and normal hepatocyte morphology (grade 0) (Fig. 6(a–b and a\–b\)).

Hepatic section of animal’s group exposed to radiation displayed lytic and pyknotic alterations in the hepatocyte nuclei. Apoptotic cells were scattered all over hepatic lobules with intracellular fat droplets in the peripheral zone. Dilatation of central vein as well as Kupffer cell hyperplasia were observed (grade IV). Masson trichrome–stained histopathological tissue section showed thickening of portal vein excess deposition of fibrous in portal triads grade (grade III) (Fig. 6(c–c\)). The IRR+MMF group showed notable advancement compared to the prior group. Hepatocyte swelling, hepatic sinusoidal constriction, and Kupffer cell hyperplasia were seen (grade I). Masson trichrome–stained histopathological tissue section showed normal histological structure and delicate fibrous tissues in portal triads (grade 0) (Fig. 6(d–d\)).

## Discussion

The liver’s wound-healing reaction to long-term damage is known as liver fibrosis, and it involves the production and remodeling of extracellular matrix, inflammation, angiogenesis, and cell proliferation. Evidence from human fibroblasts and fibrosarcoma cells indicates that early growth response 1 is involved in fibrogenesis since it promotes growth factor transcription, reactive oxygen species generation, and collagen production (Alexander et al. 2002; Pritchard and Nagy 2010; Bhattacharyya et al. 2011; Pritchard and McCracken 2015). Additionally, it boosts TGF-β, PDGF, along with other fibrogenic growth factors and cytokines (Jimenez et al. 2004; Khachigian 2023). TGF-β, an upstream regulator of Egr-1, exerts an important role in controlling fibrogenesis (Chen et al. 2006). Each of these factors contributes to the development and persistence of fibrosis. Indeed, the existence of the “Egr-1 signature” may predict more rapid fibrosis advancement (March et al. 2009).

This research provides a new understanding of the molecular mechanisms behind MMF’s hepatoprotective and antifibrotic effects in liver fibrogenesis caused by gamma irradiation. Such is made possible by a deeper comprehension of the Egr-1 involvement. Western blot analysis demonstrated that irradiated rats exhibited a marked elevation of Egr-1, α-SMA, TGF-β, and PDGF protein expression, in addition to elevated hepatotoxicity, disrupted oxidative stress, and elevated hepatic inflammatory mediators in their tissues. These results are in accordance with El-Shawi et al. (2023) who reported that radiation activated and proliferated HSCs, and greatly enhanced the expression of the profibrogenic cytokines TGF-β and PDGF levels.

Treatment with MMF repressed Egr-1 signaling induced by IRR. In the same concern, Huang et al. (2018) and Zhang et al. (2022) reported that MMF substantially prevented HSC activation and generated an enormous reduction in the production of proteins associated with fibrosis. MMF reduces fibroblast activity and collagen formation (Scheel et al. 2007; Scheel Jr et al. 2012). Since TGF-β is essential for fibroblasts to produce collagen, MMF’s stimulation of fibroblast reduction may help halt the fibrotic process (Hussein et al. 2015).

This study’s findings suggest that irradiated rats (IRRs) showed a notable decline in body mass gain in comparison to the control rats. Studies by Moccia et al. (2010) and El Shawi et al. (2015) have extensively documented similar decreases in body mass brought on by radiation. After radiation exposure, animals may have biphasic alterations, which could indicate serious toxicity as indicated by weight loss. Losing weight in the initial stage may be attributed to gastrointestinal damage caused by irradiation, whereas the following stage correlates to reduced water intake and a higher rate of catabolic processes (Bond and Sugahara 1969; Moccia et al. 2010). Furthermore, the IRR group’s elevated liver mass index (%) in contrast to the control group suggests progressive hepatic injury in the irradiated rats. Our outcomes are consistent with earlier research (Pradeep et al. 2008; El Shawi et al. 2015). The administration of MMF to irradiated rats, as seen in the IRR+MMF group, improved many alterations reported in the IRR group, suggestive evidence that MMF was hepatoprotective and decreased radiation-induced liver damage. This research found that the IRR+MMF group had a significantly higher body mass and a lesser liver mass index than the IRR group. These findings are consistent with Binuclara et al. (2013). This outcome, which highlights the hepatoprotective effects of MMF, could be explained as the restoration of nutritional absorption and liver homeostasis following MMF treatment.

This investigation revealed an alarming rise in hepatotoxicity indicators (AST, ALT, and GGT combined with significant reduction in TP) in serum from the IRR group compared to the control group. The upward trend in liver function parameters in gamma irradiated rats is consistent with Ibrahim (2011) and El Shawi et al. (2015). Damage to hepatocytes’ cellular membranes may be the cause of permeability increases and facilitates the cytoplasmic enzymes to pass outside the cells, raising serum aminotransferase activity (Gaur and Bhatia 2009). In addition, TP is an accurate clinical indicator of hepatic synthetic function (Akter et al. 2021). Consistent with various findings (Navarro and Senior 2006), our findings showed that inducing liver fibrosis in rats resulted in a considerable diminution in total protein, indicating additional liver damage (Zhang et al. 2015). However, administering MMF to irradiated rats reversed the hepatotoxicity caused by irradiation, implying that MMF’s hepato-protective efficiency and membrane stabilizing efficacy (Ferjani et al. 2016) prompted the formation of new liver cells and hastened the process of regeneration by stimulating protein synthesis.

The formation of ROS exacerbates oxidative stress, which is a major factor in the development of hepatic failure. It also heightens the inflammatory response, which in turn triggers the synthesis of profibrogenic mediators and starts the process of hepatic fibrogenesis (Cui et al. 2003; Galli et al. 2005; Ghiassi-Nejad and Friedman 2008). ROS are a key fibrogenic trigger that can induce liver fibrosis by encouraging the overproduction of ECM, cell proliferation, and HSC activation (Henderson and Iredale 2007). Therefore, diminishing ROS to mitigate oxidative stress is a realistic goal for liver fibrosis management (Gu et al. 2016). The present research revealed that radiation caused a noteworthy drop in oxidative stress indicators, as shown by crucial drops in Nrf-2, HO-1, and GPx activities combined with elevated activity of NOX-4 and NO level. These results are in accordance with Sinha et al. (2012), Li et al. (2015), Abdel‐Magied and Shedid (2019), and Eassawy et al. (2021).

The disruption of the Nrf2 system following γ-radiation exposure may be related to ROS activation, which promotes antioxidant response element (ARE)–dependent gene expression. However, as ROS levels increase, Nrf2 levels fall. This is followed by a decrease in Gpx and HO-1 activity (Kang et al. 2004). The most essential Nrf2 target gene is HO-1. It is a cytoprotective antioxidant enzyme that can depress the oxidative stress. HO-1 metabolizes heme, producing iron, carbon monoxide, and biliverdin, which is transformed into bilirubin by biliverdin reductase. These products are potent antioxidants that fight oxidative stress (Osman et al. 2016).

Lan et al. (2015) demonstrated the significant impact NOX-4 plays on liver fibrosis, mostly via activating HSCs directly. Likewise, NO, an essential component of nitrosative pro-oxidants and a modulator of systemic vasodilatation, was found to be raised in hepatic cirrhosis patients. Additionally, NO was linked to acute liver damage generated by carbon tetrachloride (CCl4) in liver fibrosis models (Mohammed et al. 2003; Tipoe et al. 2006). Eventually, the reduction of GPx activity increases the susceptibility of hepatic tissue to oxidative stress shocks and is crucial for cellular defense against oxidative stress and cellular damage (Ramos et al. 2014; Louvet and Mathurin 2015). Interestingly, administering MMF to the irradiated rats revealed significant reductions in hepatic NOX-4 activity and NO, as well as boosted antioxidant defenses in Nrf-2, HO-1, and GPx activities in hepatic tissue compared to the irradiated group. These outcomes are in agreement with Ferjani et al. (2016) who have proven MMF’s antioxidant potential.

Chronic inflammation promotes ongoing hepatocyte destruction and, eventually, hepatic fibrosis (Hussain 2009). After liver injury, activated hepatic Kupffer cells release pro-inflammatory mediators and chemokines which stimulates the circulating immune cells to participate in the response to inflammation (Xiao et al. 2012). There’s a connection between oxidative stress and inflammation, though, as the antioxidant system is needed to lessen the liver inflammatory response (Campo et al. 2008). This study found that pro-inflammatory mediators such as NF-_K_B, TNF-α, and IL-6 were significantly higher in irradiated rats’ livers compared to control rats these findings are consistent with Luedde and Schwabe (2011) who showed that ROS-induced activation of cytoplasmic NF-_K_B leads to the generation of cytotoxic cytokines, including tumor necrosis factor-α then the additional mediators of inflammation become active, leading to an overexpression of IL-1 and IL-6 (Ali et al. 2015).

However, administering MMF reduced their expression significantly. Concerning the same issue, Huang et al. (2018) mentioned that MMF treatment reduced considerably cytokine levels and mRNA expression in liver tissue, specifically TNF-α and IL-6. TNF-α can trigger proinflammatory agents such as NO, IL-1, IL-6, and IL-8, leading to further liver injury. MMF treatment diminishes both the beneficial feedback loop between inflammation and ROS as well as the hepatic local inflammatory response (Tülek et al. 2000; Nadler et al. 2001).

The liver sections of the irradiated group presented significant pathological abnormalities, as shown by the histological examination. These outcomes of radiation were confirmed by Meydan et al. (2011), El Shawi et al. (2015), and El-Shawi et al. (2023). The typical architecture of the hepatic lobule was evident in liver sections from the MMF group. Administering MMF to the irradiated rats lessened the alterations caused by IRR. This implies that MMF has hepatoprotective properties, which is consistent with the observations of Yang et al. (2011) and Huang et al. (2018). Masson’s trichrome staining of liver sections from the IRR+MMF group revealed anti-fibrotic characteristics in comparison to those from the IRR group, further demonstrating that MMF treatment reduced hepatic collagen deposition levels. As radiation therapy remains an essential strategy in the treatment of numerous cancers, MMF may be a useful supplement therapy to reduce subsequent harm to the liver, increasing patients’ overall quality of life and long-term prognosis. Despite the promising outcomes, this study has limitations. Because most of the data may come from preclinical models, they may not fully reproduce the complexities of human liver fibrosis. To overcome this, future studies should include clinical trials with a wide range of patient populations to evaluate the findings’ applicability.

Overall, this study emphasizes MMF’s hepato-protective and anti-fibrotic properties against radiation-induced liver fibrosis. As Egr-1 seems to operate as a “director” of the intricate fibrosis symphony, focusing on Egr-1 expression or activity may be a novel approach to controlling fibrosis in a subgroup of patients and preventing pathological fibrogenesis.

## Data Availability

All source data for this work (or generated in this study) are available upon reasonable request.
